# Attachment networks in young adults

**DOI:** 10.3389/fpsyg.2023.1321185

**Published:** 2024-02-06

**Authors:** Lucia L. Carli, Paolo Alessandro Alì, Elena Anzelmo, Claudia Caprin, Franca Crippa, Marcello Gallucci, Loredana Moioli, Daniela Traficante, Judith A. Feeney

**Affiliations:** ^1^Department of Psychology, University of Milan-Bicocca, Milan, Italy; ^2^Department of Psychology, Università Cattolica del Sacro Cuore, Milan, Italy; ^3^School of Psychology, The University of Queensland, Brisbane, QLD, Australia

**Keywords:** attachment network, young adults, attachment functions, attachment strength, primary figure, full-blown attachment

## Abstract

**Introduction:**

This study investigated attachment networks in a sample of Italian young adults. Attachment networks were defined in terms of attachment functions, attachment strength, the presence of a primary figure, and full-blown attachments.

**Method:**

Participants were 405 young adults, and we studied the effects of the demographic variables of gender, romantic status (whether single, involved in a romantic relationship for less or more than 24 months) and employment (whether university students or workers) on the structure of attachment networks. Participants were asked to answer the WHO-TO questionnaire, and derived indexes were analyzed using mixed ANOVAs, linear and logistic regression techniques.

**Results:**

Results indicated that while friends still had great importance in the network, partners were acquiring increasing relevance; at the same time, parents, and particularly mothers, remained central figures, particularly for the secure base function. Regarding the demographic variables, we observed that women reported stronger bonds with their mothers than men did, while the importance of friends was higher for men than for women. Additionally, our study supports previous findings underlining the importance of romantic partners in this phase of life, with participants involved in romantic relationships for longer than 24 months showing a fully developed attachment bond with their partners. Finally, for workers, the transfer of functions from the family-of-origin to external figures seemed to be fostered.

**Discussion:**

In conclusion, young Italian young adults go through a phase of intensive restructuring of attachment bond networks, particularly in relation to the consolidation of romantic relationships and work commitments.

## 1 Introduction

Bowlby hypothesized that attachment bonds, although investigated primarily in infancy, are crucial throughout the whole lifespan (Bowlby, 1969, 1973, 1980). Across the life-cycle, attachment bonds maintain the same four functions (Bowlby, 1982; Weiss, 1991): proximity seeking (assuring closeness to the target figure); separation protest (experiencing distress when that figure is absent); safe haven (relying on protection and support of the target figure in stressful situation); and secure base (moving away from the target figure to explore novel environments).

Although attachment bonds serve similar functions throughout the lifespan, over time individuals develop attachment bonds with new figures. Infants develop their first attachment bonds with their caregivers. Later, peers and then romantic partners gradually acquire greater relevance and become additional attachment figures (van IJzendoorn et al., 1992; Doherty and Feeney, 2004). The process through which the relationship with a relevant figure becomes an attachment bond has been investigated: the four functions are gradually assigned to a target figure, beginning with proximity-seeking and ending with secure base. A relationship can be defined as a full-blown attachment only when this process is complete (Hazan and Zeifman, 1994; Fraley and Davis, 1997).

Since individuals may have multiple attachment figures, we can identify a network of attachment bonds for each person; each figure can be more or less relevant for the individual, fulfilling each function to varying degrees (Umemura et al., 2018a; Carli et al., 2019). Within this theoretical framework, two other concepts are usually investigated: primary figures and full-blown attachments. Primary figures are those characterized by the highest attachment strength (the highest composite rank for attachment strength across the four functions); as noted above, full-blown attachments are figures fulfilling all the functions, although not necessarily being highest in terms of attachment strength (Feeney, 2004).

The extending of attachment networks to figures outside the family of origin occurs particularly during adolescence and young adulthood (Ainsworth, 1989). The importance of young adulthood as a transition phase has been recognized by McGoldrick and Carter (1982): young adults usually start to differentiate themselves from the family of origin, defining their future career and sexual life, while developing new and significant relationships gradually characterized by higher levels of mutual care and trust.

The duration of this phase has extended progressively since the late 80s (Cherlin et al., 1997), with young adults now remaining longer in their family-of-origin home, and still economically and affectively dependent upon their parents. This trend applies to both men and women, even though with some differences (Markiewicz et al., 2006; Eurostat, 2022): in particular, in the European Union, it seems that women leave their family-of-origin homes consistently earlier than men.

Further, although the trend towards a longer period of economic and emotional dependence may be particularly true for Mediterranean countries, it has been observed in other countries as well (Scabini and Donati, 1989; Goldscheider, 1997; Scabini and Cigoli, 1997). This social trend has co-occurred with profound changes in gender roles observed in Western societies since the 1970s. These changes have impacted on the relationships between young adults and their family-of-origin; they have also impacted on the formation of couple relationships and new family units (CISF – Centro Internazionale Studi Famiglia, 2020, 2021) and on the extent of conflict within couples (Riva et al., 2020).

Several factors have shaped this trend in Italy. Financial and economic crises have made it harder for young adults to attain independence and leave their family home. Furthermore, the shift from a “normative” to an “affective” family (Pietropolli Charmet, 2000; Pietropolli Charmet and Savuto, 2001; Gambini, 2011; Benzoni, 2017) has reduced the distances between family roles. Parents’ increased focus on preserving their children’s happiness and fulfilling their wishes has led to friendlier relationships, but weakened parental authority. Children may be granted extensive freedom from an early age; parents in affective families may over-emphasize their children’s abilities, and struggle to support offspring through key developmental challenges. In this context, offspring may become hesitant about their imperfections, failures, and future plans (Gambini, 2011; Bernardini, 2012; Benzoni, 2017; Lancini, 2023). These family attitudes align with the socio-cultural model based on the myth of eternal youth (Bauman, 2000, 2003, 2009), which discourages definitive choices and inhibits identity construction. The restructuring of family and non-family attachments in young adulthood is understudied in this new social reality of delayed independence, and prolonged romantic and occupational decision-making.

The present study aims to better understand the attachment bonds of young adults, in the context of these important social and relational changes. In particular, we aim to extend our understanding of the positioning of relevant attachment figures within the network hierarchy. Our study also aims to clarify how gender, romantic status and employment – both independently and in combination – can affect the structure of the attachment network.

Therefore, in this work, we analyze attachment networks in a group of young adults who differ in romantic status (single, or involved in a romantic relationship for less than or more than 24 months) and employment status (working or studying), as well as gender. Specifically, our aims are to:

1. investigate the composition of attachment networks in the full sample of young adults. For this goal, we studied the reliance on each significant figure for each function, and overall attachment strength; additionally, we examined the presence of primary figures and full-blown attachments. In relation to this aim, we hypothesize that friends are crucial figures within the network, particularly for the safe haven function, while partners are expected to acquire greater relevance in the network (Hypothesis 1). We further hypothesize that these changes impact only partially on the relevance of mothers, with mothers still being central for the secure base function (Hypothesis 2);
2. verify if the composition of attachment networks differs according to romantic status, employment status, and gender; and to examine possible interactions between romantic status, employment status and gender. In relation to this aim, we hypothesize that both the presence and the duration of a romantic relationship is associated with greater relevance of romantic partners across functions (Hypothesis 3). Further, we hypothesize that, compared to students, young workers show greater reassignment of attachment functions to figures external to the family of origin, and in particular, to romantic partners (Hypothesis 4).

## 2 Materials and methods

### 2.1 Participants

The research involved 405 Italian participants, (*M* age = 21.23, *SD* age = 1.52); 183 participants were men and 222 were women. Of the total, 202 were workers (49% of the men, 51% of the women), while 203 were students (51% of the men, 49% of the women). Students were enrolled at several faculties of three main universities of Milan (University of Milan-Bicocca, State University of Milan, and the Polytechnic University of Milan). Specifically, notices were posted on bulletin boards of the university campuses; additionally, students were informed about the study by professors at the beginning of the classes. Students were sampled equally from the Science and Humanities departments. Workers were recruited by means of snowball sampling: five researchers (seed units) started the process, and the waves were propagated until the number of workers matched the number of students.

Students’ mean age was 20.86 (*SD* = 1.43), while workers’ mean age was 21.61 (*SD* = 1.53). More specifically, within the group of students, mean age of men was 21.20 (*SD* = 1.40), and mean age of women was 20.56 (*SD* = 1.40); among the workers, mean age of men was 21.78 (*SD* = 1.62), and mean age of women was 21.48 (*SD* = 1.45).

Most of the students held high-school diplomas and were enrolled in their Bachelor Degree (88% of men and 93% of women), while the remaining were enrolled in their Master Degree. Among the workers, the percentage of participants with a high-school diploma was 78% among men and 74% among women; 15% of men and 13% of women achieved just a middle-school diploma. Most of the workers were either technicians (22% of men, 15% of women), employed in administration or service-related activities (34% of men, 75% of women) or tradespersons and laborers (37% of men, 7% of women).

Participants who were in a romantic relationship were divided into two groups, defined by relationship length of more than or less than 24 months, since research suggests that attachment bonds are usually fully established within 24 months (Chris Fraley and Davis, 1997; Trinke and Bartholomew, 1997). Among the full sample, 173 (43%) had been single, 110 (27%) were in a relationship for less than 24 months, and 122 (30%) were in a relationship for more than 24 months. Regarding the student sample, 67% of men and 36% of women were single, and overall, women had been engaged in romantic relationships for longer than males (17% of men and 35% of women were in a relationship for longer than 24 months); the remaining 16% of men and 29% of women were involved in a relationship for less than 24 months. Among the workers’ sample, we observed more homogeneity: 40% of men and 31% of women were single, and, among those in a relationship, 30% of men and 36% of women had been in a relationship for longer than 24 months; 30% of men and 33% of women were involved in shorter relationships.

Since heterogeneity of groups could affect the results, we tested whether there were significant differences between men and women with regards to age, employment status and romantic status. Using independent sample *t*-tests, we found a significant difference with regards to age (*t*(403) = 3, *p* = 0.003), with men being significantly older (*M* = 21.48, *SD* = 1.53) than women (*M* = 21.03, *SD* = 1.49), although with a small effect size (*Cohen’s d* = 0.3; Cohen, 1988).

To investigate gender differences in the categorical variable of employment status, we used the Chi-square independence test in order to test the null hypothesis of independence of the two frequency distributions (McHugh, 2013; Nihan, 2020). The test was not significant ($\chi^{2}$(1) = 0.12, *p* = 0.72), hence employment status was independent of gender: the ratio of working men (51%) and the ratio of working women (49%) were similar. Additionally, we tested the relationship between employment status and romantic status (being single vs. being in a romantic relationship). This test was significant ($\chi^{2}$(1) = 9.48, *p* = 0.002), with the ratio of workers involved in a romantic relationship (57%) being significantly higher than the ratio of students involved in a romantic relationship (43%).

For romantic status, we firstly ran a Chi-square test to investigate gender differences between single participants and those in a romantic relationship (as a whole): the test proved significant ($\chi^{2}$(1) = 16.84, *p* < 0.0001); in our sample, a higher percentage of women (67%) than men (46%) were in a relationship, with a medium effect size (*Cramer’s V* = 0.20). A Chi-square test regarding relationship length and gender was not significant ($\chi^{2}$(1) = 0.03, *p* = 0.85), indicating that the ratio of men in longer relationships (over 24 months) was similar to the ratio of women in longer relationships (52% of the men, 58% of women).

### 2.2 Procedure

The study received approval by the Institutional Review Board of the University of Milan-Bicocca. All participants agreed to participate, providing written informed consent after being informed about the aims of the research design.

Each subject completed a socio-demographic form and a questionnaire regarding their attachment network (30 min on average were needed for completion of both). A research assistant provided all the information needed prior to the beginning of the study, and was available for any further questions during the questionnaire completion phase. Once the questionnaire was completed, in order to maximize confidentiality, each participant was asked to insert the sheets in an envelope which was then collected by the researchers.

### 2.3 Measures

Participants were presented with an Italian version of the WHO-TO scale, already adopted for a similar study investigating attachment networks in committed couples (Carli et al., 2019). The WHO-TO was initially developed by Hazan and Zeifman (1994) as a 12-item interview, investigating the four attachment functions (see below) using three questions each. Doherty and Feeney (2004) developed the English self-report version of the instrument by using only eight items (two per function).

The Italian self-report version mirrors the original structure of the interview, including 12 items. Specifically, each participant was asked to name up to five attachment figures he or she would turn to for Proximity Seeking (e.g., “Who is the person you most like to spend time with?”), Separation Protest (e.g., “Who is the person you do not like to be away from?”), Safe Haven (e.g., “Who is the person you would count on for advice?”) and Secure Base (e.g., “Who is the person you can always count on?”), by listing them in order of importance. For each of the 12 items, in line with the scoring approach used by Doherty and Feeney, each figure was assigned a score from 5 (the first named) to 1 (the last named). *Reliance* on a specific figure for a specific attachment function was computed by summing the scores across the three items for that function (total ranging from 1 to 15). If the same type of figure was named more than once for the same item, only the first mention was assigned a score.

In order to study the relative importance of each figure within the network, three other indexes were defined. *Attachment strength* (ranging from 1 to 15) is obtained by computing the mean reliance on a specific figure across each function; *full-blown attachment* is present when a figure scores at least 8 for each function, with the total attachment strength for that figure greater than, or equal to 32; a *primary attachment figure* is the one with the highest score across all the functions. Although our indexes are analogous with those used by Doherty and Feeney (2004), those researchers did not consider separation protest when computing full-blown attachment. Both full-blown attachment and primary attachment figures were coded as dichotomous variables, with 1 indicating that the figure satisfied the criteria and 0 that they did not.

The prior Italian study on committed couples showed the instrument to have good reliability for the four functions, with alpha coefficients ranging from 0.84 to 0.92 (Carli et al., 2019). In the current study of young adults, alpha coefficients for the four attachment functions ranged from 0.71 for friends to 0.96 for partners, again demonstrating adequate reliability.

### 2.4 Data analysis

#### 2.4.1 Overall attachment networks

Regarding our first goal, investigating attachment networks in the overall sample, we assigned all the named attachment figures to one of 16 coding categories. These 16 categories were then reduced to a smaller number to simplify data analysis. This process involved checking the frequency of occurrence of each category, and grouping any categories exhibiting a minimal frequency into broader classes (e.g., since all relatives except siblings and parents were cited rarely, we created a wider class of “relatives”).

Subsequently, we computed descriptive statistics for reliance and attachment strength for each target category, and tested whether the observed differences were significant across targets by using a set of Repeated-Measures ANOVAs with Huynh-Feldt correction. The analyses were carried out with the R Package rstatix (Kassambara, 2020). Then, for each target category, we investigated the observed frequencies of primary figures and full-blown attachments.

#### 2.4.2 Networks as a function of gender, romantic status, and employment

Regarding the second goal, the study of attachment networks among participant subgroups, we firstly explored the effects of gender, employment status and romantic status on the mean reliance scores for each function and on attachment strength. We used a set of repeated-measures ANOVAs with Huynh-Feldt corrections (if needed in case of violations of the sphericity assumption). The first four analyses were run for each attachment function, with reliance as dependent variable, and target figures and the demographic variables as independent variables. The fifth analysis was run with the same independent variables, but with attachment strength as dependent variable. These initial analyses were also repeated adding age as a covariate, in order to check if age had any significant impact on its own or in interaction with other variables. Since age did not exert any significant impact, it was dropped from the analyses.

The repeated-measures analyses checked whether reliance or strength scores varied depending on the target figures and on the demographic variables at a general level (in the overall sample). Then, we investigated the effects of our variables more specifically, by examining which subgroup of participants had significantly higher mean scores (for each target figure, within each function). Accordingly, each repeated-measures analysis was followed by a set of univariate ANOVAs: the dependent variables were each target figure score, and independent variables were those found to be significant in the respective repeated-measures analysis. For the univariate analyses we used the function lm of the R package stats (R Core Team, 2019). Significant relationships were further investigated using the R package emmeans (Lenth, 2020). Specifically, we employed simple effect analysis to study two-way interactions, and simple interaction analysis to analyze three-way interactions, when present. For both type of interactions, if more than two levels were involved, we used post-hoc analyses and employed the Bonferroni correction for *p*-values adjustment.

Lastly, we investigated whether our demographic variables were associated with having a given primary attachment figure or full-blown attachment, using Multinomial Logistic Regression. This technique first provides an omnibus test, to verify which demographic variables have a significant impact on the dependent variables. Then, it examines the differences in the likelihood of having a given target figure as the primary figure or as full-blown attachments, by investigating pairwise comparisons among the subgroups of interest (Little, 2013). For each significant difference we used the Odds Ratio (OR) and the respective confidence intervals, to give an estimate of the effect size (Chu et al., 2020). For these analyses, we used the function multinom of the R package nnet (Venables and Ripley, 2002).

Given the relatively large number of analyses in our study, and the importance of reducing the likelihood of Type 1 errors, we adopted a more conservative threshold for significance for all results, including only those with value of *p* equal to or less than .01.

## 3 Results

### 3.1 Overall attachment networks

Based on frequency analyses of the 16 identified target figures, these were clustered into six main target categories: father, mother, siblings, partner, friends, and relatives (other than parents and siblings). Table 1 shows the descriptive statistics for reliance on each figure for each function, together with overall attachment strength to each figure.

**Table 1:** Mean and standard deviation for each target figure, regarding reliance for each attachment function, and for attachment strength.

Target categories	Proximity seeking		Separation protest		Safe haven		Secure base		Attachment strength	
	*M*	*SD*	*M*	*SD*	*M*	*SD*	*M*	*SD*	*M*	*SD*
Partner	8.33	6.77	7.84	6.72	6.87	6.34	5.01	5.51	7.01	1.47
Mother	2.62	3.48	5.00	5.13	6.60	5.29	9.65	5.68	5.97	2.95
Father	1.40	2.51	2.95	4.06	2.81	4.12	6.65	5.59	3.46	2.24
Siblings	2.08	3.51	2.46	4.17	2.11	3.70	3.04	4.38	2.42	0.45
Friends	11.26	3.67	5.58	5.28	10.08	4.60	6.17	4.92	8.27	2.82
Relatives	1.92	3.35	1.70	3.47	1.45	3.23	2.61	4.47	1.92	0.50

Repeated measures ANOVA confirmed the presence of significant differences across figures for all attachment functions, as seen in Table 2.

**Table 2:** Results of repeated measures ANOVAs for each attachment function and for attachment strength.

	*F*	df1	df2	*p*	$\eta^{2}$
Proximity seeking	392.28	2.92	1178.29	<0.001	0.46
Separation protest	88.62	3.82	1543.76	<0.001	0.31
Safe haven	201.09	3.47	1401.43	<0.001	0.16
Secure base	95.83	3.84	1552.47	<0.001	0.18
Attachment strength	161.86	3.49	1409.86	<0.001	0.26

Specifically, regarding Proximity Seeking, the most highly relied on figures were friends (11.26), followed by partner (8.33). All other figures scored much lower. Post-hoc analyses showed that reliance on friends was significantly greater than reliance on partners (*p* < 0.0001); furthermore, both were significantly higher than all other targets (*p* < 0.0001). Friends and partners were followed by mothers and siblings, who did not differ statistically from each other, but both were significantly higher than relatives and fathers (all *p* < 0.0001).

For Separation Protest, the most highly relied on figure was the partner (7.84), followed by friends (5.58); post-hoc analyses showed that the difference between these two targets was statistically significant (*p* < 0.0001). Furthermore, reliance on friends and on partners was significantly higher than on all other figures (*p* < 0.0001), except for mothers (5.00), whose score was not statistically different from that for friends (5.58). Partners, friends and mothers were followed by fathers and siblings, who did not differ from each other. Relatives were named least for this function: their scores were significantly lower than for father (*p* = 0.0001), but did not differ from siblings.

Regarding Safe Haven, the most important figure was friends (10.08). Post-hoc analyses showed a significant difference between friends and all other figures (*p* < 0.0001). Safe haven to mother (6.60) and to partner (6.87) differed significantly from all other figures, but not among themselves. Partners and mothers were followed by fathers and siblings, who did not differ from each other. Finally, relatives scored lower than fathers (*p* < 0.0001), but not lower than siblings.

For Secure Base, the most important figure was the mother (9.65), who scored significantly higher than all other figures (each *p* < 0.0001). Fathers and friends were next in importance (and did not differ from each other), and reliance on fathers was significantly different than all the remaining others (all with *p* < 0.0001, except for the difference between father and partner, with *p* = 0.001); reliance on friends differed from all the others (all with *p* < 0.0001), except for partners. Siblings and relatives scored significantly lower than all other targets (all with *p* < 0.0001), but did not differ from each other.

Regarding overall attachment strength, the difference between figures was again significant (see Table 2). Post-hoc analyses with Bonferroni correction revealed that attachment to friends (*M* = 8.27, *SD* = 3.64) was stronger than to all other figures (partner, *M* = 7.02, *SD* = 5.93, *p* = 0.02; mother, *M* = 5.97, *SD* = 4, *p* < 0.0001; father, *M* = 3.45, *SD* = 3.31, *p* < 0.0001; siblings, *M* = 2.42, *SD* = 3.41, *p* < 0.0001; relatives, *M* = 1.92, *SD* = 3.09, *p* < 0.0001). Friends were followed by partners, who did not score significantly different than mothers (*p* = 0.06). Attachment to partner and to mothers was significantly stronger than attachment to fathers (both *p* < 0.0001). The least important figures in terms of attachment strength were siblings and relatives, who differed statistically from fathers (both *p* < 0.0001), but not from each other.

Regarding primary attachment figures, 394 participants (97.28%) had one primary figure, while 11 participants (2.72%) had two “primary” figures (figures with the same score). Among the former, 165 (41.88%) had their partner as this figure, while 133 participants (33.76%) named their friends, 64 (16.24%) their mothers, 17 (4.31%) their siblings, 9 (2.28%) relatives and 6 (1.52%) their fathers.

Regarding full-blown attachments, only 59% (240) of participants were shown to have one. Among these, 39% (94) showed full-blown attachment towards the partner and 39% towards a friend. Mothers followed next, constituting 14% of the sample (34 participants). Observed frequencies for all the other figures were notably lower (4% siblings, 3% father, 1% relatives).

### 3.2 Networks as a function of gender, romantic status, and employment

For this second goal, we first present results for reliance scores and attachment strength for each function; then we present results for primary figures and full-blown attachments. Since the analyses for the relative ordering of each figure (for reliance and attachment strength) are extensive, they are used in the discussion section only to complement the results of the univariate ANOVA where the demographic variables were used as independent variables. Detailed results and reports can be found in Supplementary material.

#### 3.2.1 Reliance score for each function

##### 3.2.1.1 Proximity Seeking

Repeated measures ANOVA for *Proximity Seeking* showed a main effect of target figures, *F*(4.25, 1671.67) = 557.99, *p* < 0.0001. Regarding the effects of our demographic variables, we found a main effect of gender, *F*(1,393) = 7.26, *p* = 0.007, and a main effect of romantic status, *F*(2,393) = 44.91, *p* < 0.0001. Furthermore, there was a 2-way interaction of romantic status (*F*(8.51, 1671.67) = 44.91, *p* < 0.0001) and gender (*F*(4.25, 1671.67) = 7.26, *p* = 0.007).

More specifically, univariate ANOVA showed a main effect of gender *F*(1,399) = 2030.16, *p* < 0.0001, and a main effect of romantic status, *F*(2,399) = 360.11, *p* < 0.0001, for proximity seeking to the partner. Women (*M* = 9.74, *SE* = 0.27) reported greater reliance than men (*M* = 8.87, SE = 0.32) on the partner for this function. Additionally, post-hoc analyses revealed, as expected, that participants in a committed romantic relationship reported greater reliance on partners than on single participants (*M* = 2.06, *SE* = 0.30). However, no significant differences were observed between individuals in a romantic relationship for more than or less than 24 months (more than 24 months: *M* = 13.35, *SE* = 0.37; less than 24 months: *M* = 12.51, *SE* = 0.39, both with *p* < 0.0001).

Proximity seeking towards the mother showed only a gender difference, (*F*(1,399) = 9.60, *p* = 0.002). Women (*M* = 3.10, *SE* = 0.23) showed higher reliance on their mothers than men (*M* = 1.99, *SE* = 0.28) for this function, *p* = 0.002.

Proximity seeking toward friends showed a main effect of romantic status, (*F*(2,399) = 33.93, *p* < 0.0001), and an interaction effect between gender and romantic status, (*F*(2,399) = 6.16, *p* = 0.002). Regarding the interaction effect, simple effect analysis showed that women in longer relationships had lower reliance on friends (*M* = 8.72, *SE* = 0.37) than women in shorter relationships (*M* = 10.65, *SE* = 0.40), *p* = 0.008, and as compared to single women (*M* = 13.32, *SE* = 0.39), *p* < 0.0001. Regarding men, there was a difference between those engaged in shorter relationships (*M* = 10.80, *SE* = 0.52) and those who were single (*M* = 12.56, *SE* = 0.33), *p* = 0.01, but no difference between those in longer (*M* = 10.79, *SE* = 0.51) and shorter relationships.

##### 3.2.1.2 Separation Protest

Repeated measures ANOVA for *Separation Protest* revealed a main effect of target figures (*F*(4.08, 1601.54) = 124.92, *p* < 0.0001). Regarding the demographic variables, we again found a main effect of gender, (*F*(1,393) = 15.53, *p* < 0.0001) and a main effect of romantic status, (*F*(2,393) = 7.67, *p* < 0.0001). Reliance score for this function also revealed two-way interactions between target figures and romantic status (*F*(8.15, 1601.54) = 54.02, *p* < 0.0001), and between target figures and employment status (*F*(4.08, 1601.54) = 4.66, *p* = 0.0003).

Univariate ANOVA of *Separation Protest* from partners revealed a main effect of romantic status, (*F*(2,393) = 239.75, *p* < 0.0001). Post-hoc tests found a significant difference between single participants (*M* = 1.92, *SE* = 0.35) and those in both shorter relationships (*M* = 11.77, *SE* = 0.45); and longer ones (*M* = 12.34, *SE* = 0.43), both with *p* < 0.0001. As for proximity seeking, no difference was found between those in shorter vs. longer relationships.

Separation protest from the mother revealed a main effect of gender, (*F*(1,393) = 11.66, *p* = 0.0007). Women (*M* = 5.80, *SE* = 0.34) reported relying on their mothers more than men (*M* = 3.95, *SE* = 0.42) for this function, similarly to the proximity-seeking function.

Separation protest from friends showed a main effect of employment, (*F*(1,393) = 15.35, *p* = 0.0001), with students (*M* = 6.48, *SE* = 0.40) reporting greater reliance on their friends than workers (*M* = 4.40, *SE* = 0.35).

Separation protest from siblings showed a main effect of gender, (*F*(1,393) = 7.81, *p* = 0.005), and a main effect of romantic status, (*F*(1,393) = 8.52, *p* = 0.0002). Regarding the former, women (*M* = 2.95, *SE* = 0.27) reported higher reliance on their siblings than men (*M* = 1.75, *SE* = 0.34). Regarding romantic status, post-hoc analysis revealed a significant difference between single participants (*M* = 3.49, *SE* = 0.32), who were the least reliant, compared to those in both shorter (*M* = 2.01, *SE* = 0.41), *p* = 0.01, and longer relationships (*M* = 1.55, *SE* = 0.39), *p* = 0.0004.

##### 3.2.1.3 Safe-Haven

Repeated-measures ANOVA for *Safe-Haven* showed a main effect of target figures (*F*(3.97, 1562.01) = 233.09, *p* < 0.0001). Regarding our demographic variables, we found significant two-way interactions between target figures and gender (*F*(3.97, 1562.01) = 4.68, *p* = 0.0003), target figures and romantic status (*F*(7.95, 1562.01) = 53.27, *p* < 0.0001), and target figures and employment status (*F*(3.97, 1562.01) = 2.98, *p* = 0.01).

The univariate ANOVAs for *Safe Haven* for partner revealed a main effect of romantic status, (*F*(2,393) = 294.61, *p* < 0.0001). Post-hoc analysis revealed that participants in shorter relationships (*M* = 10.06, *SE* = 0.40) relied more on the partner than single participants (*M* = 1.21, *SE* = 0.31), *p* < 0.0001, but less than those in longer relationships (*M* = 12.09, *SE* = 0.38), *p* = 0.0008.

Safe haven to friends showed a main effect for each demographic variable; specifically, gender, (*F*(1,393) = 7.15, *p* = 0.008), employment, (*F*(1,393) = 10.11, *p* = 0.002), and romantic status, (*F*(2,393) = 14.96, *p* < 0.0001). Regarding gender, women (*M* = 10.51, *SE* = 0.29) relied more on friends for this function than men (*M* = 9.27, *SE* = 0.36). Regarding employment status, students (*M* = 10.63, *SE* = 0.35) reported relying more on friends than workers did (*M* = 9.15, *SE* = 0.31). Post-hoc analysis regarding the effect of romantic status revealed that participants in longer relationships (*M* = 8.26, *SE* = 0.42) relied less on friends than both participants in shorter relationships (*M* = 10.19, *SE* = 0.44), *p* = 0.005, and single participants (*M* = 11.22, *SE* = 0.34), *p* < 0.0001.

Safe Haven to fathers showed only a main effect of gender, *F*(1,393) = 10.73, *p* = 0.001. Specifically, men (*M* = 3.63, *SE* = 0.34) reported greater reliance than women (*M* = 2.21, *SE* = 0.27) on their fathers for this function.

##### 3.2.1.4 Secure Base

Repeated-measures ANOVA for *Secure Base* indicated a main effect of romantic status on reliance scores, (*F*(2,393) = 12.10, *p* < 0.0001). Besides a main effect of target figures (*F*(3.58, 1408.12) = 97.35, *p* < 0.0001), there were two-way interactions between target figures and employment (*F*(3.58, 1408.12) = 3.71, *p* = 0.002), and target figures and romantic status (*F*(7.17, 1408.12) = 22.26, *p* < 0.0001). In addition, there was a three-way interaction between target figures, gender, and employment (*F*(3.58, 1408.12) = 3.53, *p* = 0.004).

Specifically, univariate ANOVA on *Secure Base* to partners showed a main effect of romantic status, (*F*(2,393) = 164.82, *p* < 0.0001). Participants in shorter relationships (*M* = 6.40, *SE* = 0.41) reported more reliance on partner than singles (*M* = 0.86, *SE* = 0.32), *p* < 0.0001; in turn, those in longer relationships (*M* = 9.69) reported more reliance on partner than those in shorter relationships, *p* < 0.0001.

Secure base to friends showed a main effect of employment (*F*(1,393) = 8.58, *p* = 0.004), and of romantic status, (*F*(2,393) = 10.53, *p* < 0.0001). Students (*M* = 6.80, *SE* = 0.38) reported relying more on friends (*M* = 5.33, *SE* = 0.34) than did workers, *p* = 0.004. Further, singles (*M* = 7.36, *SE* = 0.37) reported relying more on their friends than participants involved in longer relationships (*M* = 4.68, *SE* = 0.46), *p* < 0.0001.

#### 3.2.2 Attachment strength

Finally, repeated measures ANOVA on *overall attachment strength* revealed a main effect of target figures (*F*(3.73, 1466.76) = 147.21, *p* < 0.0001), as well as significant interactions between target figures and romantic status (*F*(7.46, 1466.76) = 8.20, *p* < 0.0001), and between target figures, romantic status and employment status (*F*(7.46, 1466.76) = 7.39, *p* < 0.0001).

Univariate ANOVA of *attachment strength* to partner revealed a main effect of romantic status, (*F*(2,393) = 18.99, *p* < 0.0001), as well as an interaction between romantic status and employment, (*F*(2,393) = 16,21, *p* < 0.0001). Follow-up analyses revealed that strength towards partners was lower for single workers (*M* = 2.25, *SE* = 0.65) than for all other groups. (Mean scores for the other groups ranged from 6.94 for students in shorter relationships, to 9.13 for workers in longer relationships, all with *p* < 0.0001).

#### 3.2.3 Primary figures

The Log-Likelihood Ratio test for the multinomial logistic regression investigating the effect of gender, employment status and relationship status on *primary attachment figures* showed main effects of gender ($\chi^{2}$(5) = 16.49, *p* = 0.005) and of romantic status ($\chi^{2}$(10) = 176.22, *p* < 0.0001). No interaction effects were significant (Table 3).

**Table 3:** Log-likelihood ratio test for the multinomial logistic regression on primary figures outcomes.

Dependent variables	LR Chi-square	Df	*p*-value
GENDER	16.49	5	0.005
EMPLOYMENT	7.53	5	0.18
ROMANTIC STATUS	176.22	10	2.2e-16
GENDER*EMPLOYMENT	4.71	5	0.45
GENDER*ROMANTIC STATUS	4.81	10	0.90
EMPLOYMENT*ROMANTIC STATUS	6.09	10	0.81
GENDER*EMPLOYMENT*ROMANTIC STATUS	3.99	10	0.95

**Figure 1:**
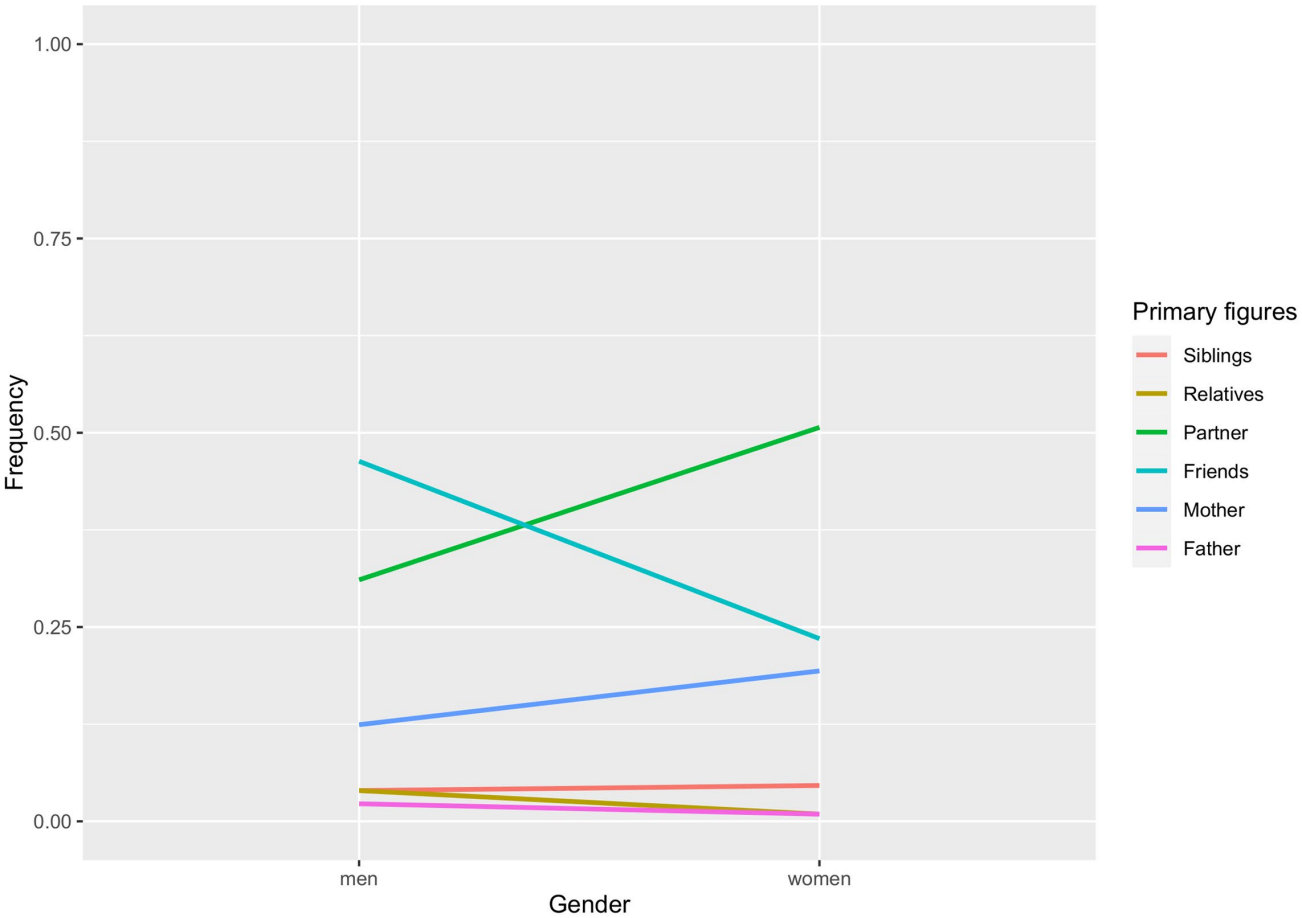
Gender differences in the likelihood for each attachment figure of being classified as a primary figure.

**Figure 2:**
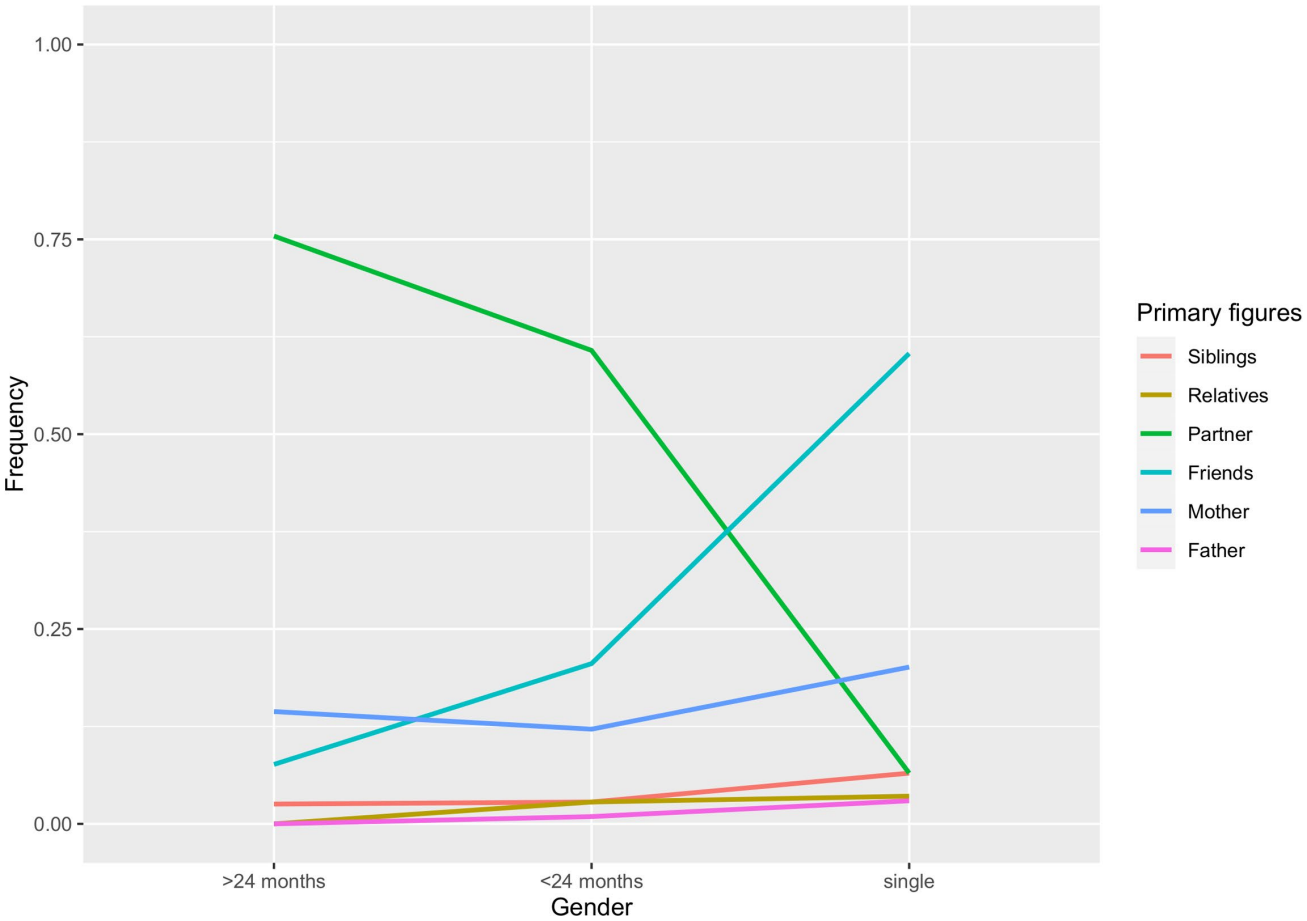
Romantic status differences in the likelihood for each attachment figure of being classified as a primary figure.

In order to interpret the results, we need to examine the relative observed frequencies for gender in Figure 1, and the respective frequencies for each level of romantic status in Figure 2. If a group shows a higher likelihood for a target figure, that does not necessarily mean that the odds for group A are higher than those for group B. Indeed, we compare the likelihood of a figure with the likelihood of a reference figure across the levels of our demographic variables.^1^

Women were 2.48 times more likely than men (*CI* = 1.35–4.56), in term of relative risk (RR), to consider their partners as the primary figure rather than their friends (*z* = 2.91, *p* = 0.004). Furthermore, women’s RR was 2.84 times more likely than men’s (*CI* = 1.50–5.37) in considering their mothers as primary figure rather than their friends (*z* = 3.22, *p* = 0.001).

Single participants’ RR was lower (*OR* = 0.040, *CI* = 0.02–0.09) in considering the partner as their primary figure rather than friends, as compared to participants involved in shorter relationships (*z* = −7.92, *p* < 0.0001); in turn, participants involved in longer relationships were 3.40 times more likely than those in shorter relationships (*CI* = 1.46–7.95) to consider their partner as the primary figure, rather than friends (*z* = 2.83, *p* = 0.005).

#### 3.2.4 Full-blown attachments

The Log-Likelihood Ratio test showed main effects of employment ($\chi^{2}$(6) = 23.29, *p* = 0.0007), and of romantic status ($\chi^{2}$(12) = 122.15, *p* < 0.001). Again, no interaction effects were significant (Table 4).

**Table 4:** Log-likelihood ratio test for the multinomial logistic regression on full-blown attachments outcomes.

Dependent variables	LR Chi-square	Df	*p*-value
GENDER	6.25	6	0.40
EMPLOYMENT	23.29	6	0.0007
ROMANTIC STATUS	122.15	12	< 2.2e-16
GENDER*EMPLOYMENT	5.25	6	0.51
GENDER*ROMANTIC STATUS	8.57	12	0.74
EMPLOYMENT*ROMANTIC STATUS	13.66	12	0.32
GENDER*EMPLOYMENT*ROMANTIC STATUS	5.63	12	0.93

**Figure 3:**
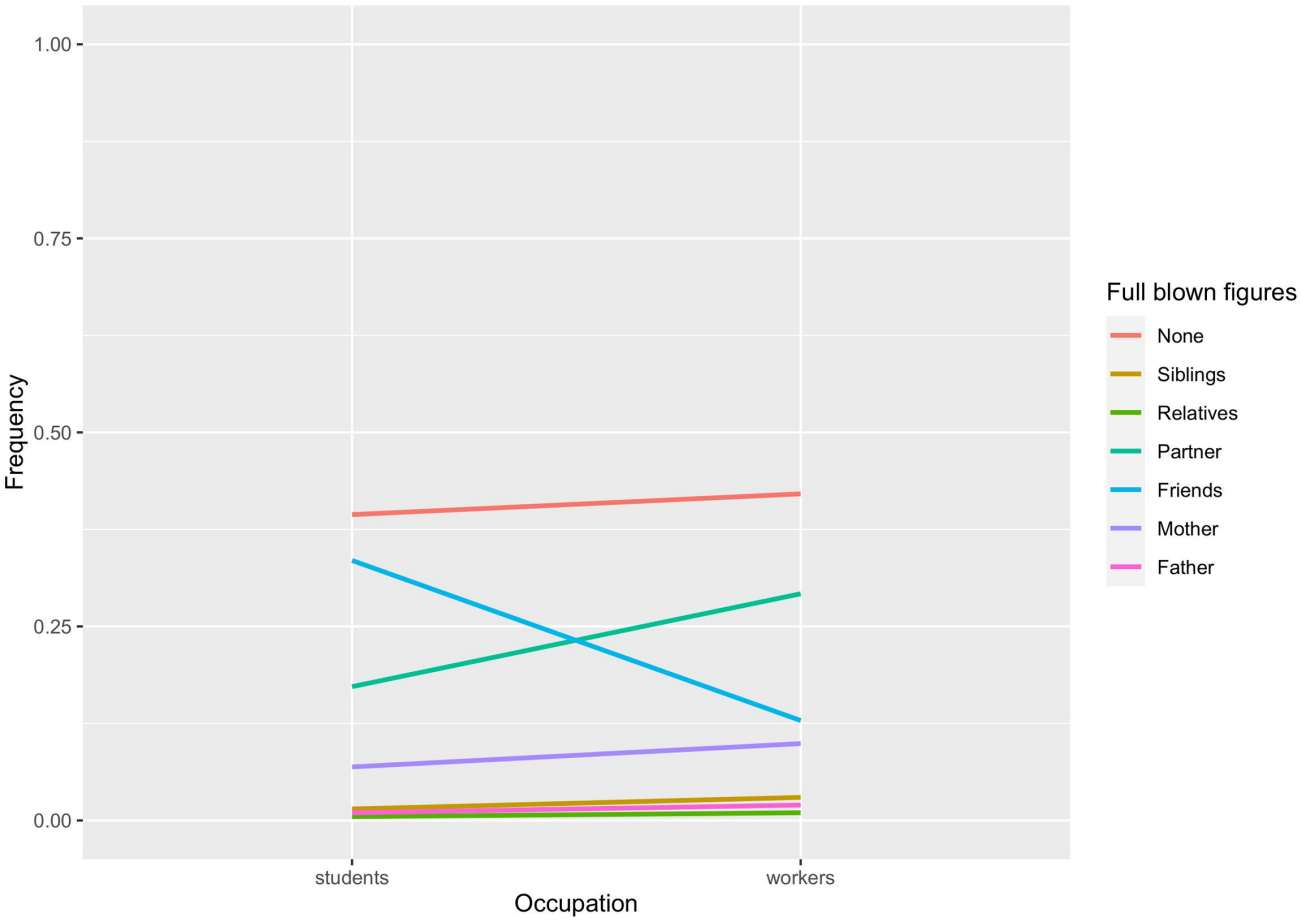
Occupation differences in the likelihood for each attachment figure of being classified as a full-blown figure.

**Figure 4:**
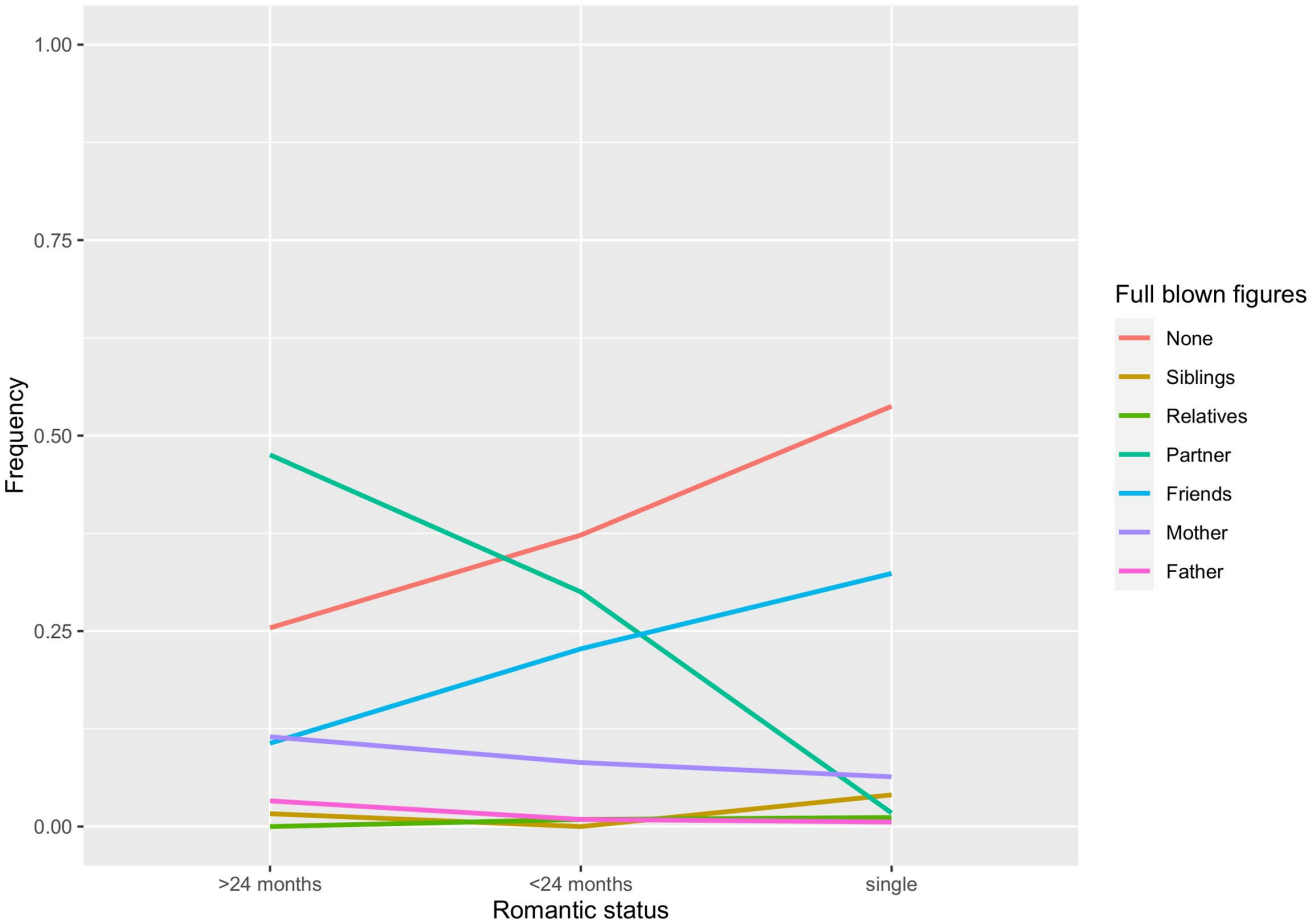
Romantic status differences in the likelihood for each attachment figure of being classified as a full-blown figure.

Figures 3, 4 show the observed frequencies for gender and romantic status. Workers ere 3.53 times more likely than students (*CI* = 1.53–8.14) to report their mothers as full-blown attachment figures rather than their friends (*z* = 2.96, *p* = 0.003). Further, workers were 3.77 times more likely than students (*CI* = 1.91–7.42) to consider their partner as full-blown attachment figure rather than their friends (*z* = 3.83, *p* = 0.0001). Workers were also 2.80 times more likely than students not to report any full-blown attachment (*CI* = 1.62–4.86), compared to the likelihood of having friends as full-blown attachments (*z* = 3.68, *p* = 0.0002).

Single participants were less likely than those in shorter relationships (*OR* = 0.048, *CI* = 0.01–0.17) to have the partner, rather than friends, as their full-blown attachment (*z* = −4.63, *p* < 0.0001). Lastly, participants in longer relationships were, in turn, 3.69 times more likely than those in shorter relationships (*CI* = 1.63–8.35) to have their partners as full-blown attachment figure rather than their friends (*z* = 3.13, *p* = 0.002).

## 4 Discussion

This research investigated attachment networks in young adults. In particular, it explored the range of emerging figures who acquire greater relevance during this phase, and who gradually replace the early attachment figures that are still present in the network, satisfying one or more attachment functions (Proximity Seeking, Separation Protest, Safe Haven, Secure Base). To further examine the transfer of attachment functions, and given evidence of young adults’ prolonged economic and emotional dependence on the family of origin, the research also explored the relationships between attachment functions and the demographic variables of gender, romantic status and employment.

### 4.1 Overall attachment networks

Young adults’ reliance on particular target figures (friends, partner, mother, father, siblings and other relatives) clearly varied across functions. Friends and partners were the most important emerging figures, consistent with Hypothesis 1. This process, already clear in adolescence (Hazan and Zeifman, 1994), proceeds during young adulthood, going alongside the formation of couple relationships and the planning of a new family unit (Carli et al., 2019). Nonetheless, during young adulthood, mothers and fathers are still important. In particular, both parental figures are very relevant for the Secure Base function, and mothers also play a crucial role for Safe Haven. These findings support Hypothesis 2. Partners are the most important figures for Separation Protest, but are relied on less than friends for both Proximity Seeking and Safe Haven. The interplay between friends and romantic partners suggests that, during this phase, partners slowly become central figures in the network, challenging the supremacy of friends in all functions but Secure Base. Siblings and relatives are the least important figures across functions.

This pattern of gradual and ongoing reassignment is consistent with the literature. Proximity Seeking and Separation Protest are the first functions to be assigned outside the family of origin, beginning in adolescence. Safe Haven, and particularly Secure Base, are usually the last functions to be reassigned (Hazan and Zeifman, 1994; Zhang et al., 2011; Heffernan et al., 2012). Furthermore, mothers and fathers are not equally important across functions, but score quite highly for some (especially Secure Base).

The greater importance of friends and partners is further evidenced by the data for attachment strength, primary figures and full-blown attachments. Recall that attachment strength is operationalized as the mean reliance across functions, primary figures as the figures most relied upon overall (across functions), while full-blown attachments figures are the ones relied on strongly for every function. These definitions explain the results we found for these composite scores (attachment strength). Friends and partners are highly relied on by participants for every function, and thus they score highly in attachment strength, and are more likely to be primary figures and full-blown attachment figures. Both mothers and fathers are highly relied upon for the Secure Base function, and to some extent for Safe Haven, but reliance on parents for Proximity Seeking and Separation Protest tends to be lower (particularly for fathers, who generally score lower than mothers). For this reason, their scores on overall attachment strength are generally low, as is their likelihood of being primary figures or full-blown attachments, with fathers being particularly less relevant in this regard than mothers.

It is worth noting that a considerable number of young adults (41% of our sample) did not show any full-blown attachment. This result can be explained by the fact that some target figures will likely come to acquire greater relevance in place of others, but that this overall process of reassignment is still in progress.

Importantly, several studies have highlighted the role of friends as transitional attachment figures, being mainly relevant in adolescence but continuing to be important in young adulthood (Hazan and Zeifman, 1994; Freeman et al., 2021). Thus, friends already begin to take the place of parental figures during adolescence, but in young adulthood, their role in turn starts to be eclipsed by newly established romantic bonds (Umemura et al., 2015, 2017). In contrast, among older adults who are in committed couple relationships, partners tend to dominate all other figures for both Safe Haven and Secure Base functions (Carli et al., 2019).

### 4.2 Attachment network as a function of gender, romantic status, and employment

In general, we observed that all our demographic variables (gender, romantic status, and employment) exerted significant effects on the reassignment of attachment functions to relevant new figures. Among the three demographic variables, romantic status is the cornerstone of attachment network restructuring, as already shown extensively in the literature (Doherty and Feeney, 2004; Hazan and Selcuk, 2015; Umemura et al., 2017). Nonetheless, both gender and employment have their role in shaping attachment networks.

#### 4.2.1 Gender

Overall, the most important attachment figures for young adults are friends and romantic partners, and this finding applies to both men and women. Nevertheless, we observed some significant gender differences.

Specifically, mothers are relied on more by women than by men across functions, except for Safe Haven and Secure Base (for which no gender difference was found). This finding is in line with a longitudinal study investigating attachment networks in a sample of Czech young adults (Umemura et al., 2018b): this study showed that overall preference for mothers was higher for women than for men across a period of two years. It is not clear whether these results reflect cultural norms (Bombi et al., 2011), or whether they can be explained by other factors. Conversely, with regard to fathers (and although fathers do not generally score high in terms of reliance), we found that men rely more on fathers than women for the Safe Haven function. This result is in line with previous research on adolescents (Markiewicz et al., 2006), and on adults in committed relationships (Carli et al., 2019).

Regarding primary attachment figures, we found two interesting gender effects. Firstly, the likelihood of having mothers as primary figures is higher in women than in men; this finding is consistent with women’s greater reliance on mothers for both Proximity Seeking and Separation Protest (as noted above). Additionally, the most likely primary figure for women is their partner, while for men it is a friend.

Consistent with the present findings from our sample of young adults, it is interesting to note that in samples of older adult couples in committed relationships, it has been observed that women consistently rely more than men on their mothers, and that friends have a greater relevance for men than for women (Carli et al., 2019). Together, these results imply that some gender differences in attachment networks are stable across these two phases of the life cycle. The previous work on committed couples in adulthood, however, revealed a gender effect for the Secure Base function, not observed in young adults: in that study, adult men rely more than adult women on their romantic partner for this function (Carli et al., 2019).

#### 4.2.2 Romantic status

Overall, romantic status had considerable impact in terms of main effects, demonstrating the important role of developing romantic relationships in this phase of life. For single participants, friends are the most important figures for all functions except Secure Base. Being involved even in a relatively short romantic relationship is enough for partners to become the highest figures in terms of reliance for Proximity Seeking and Separation Protest; analogously, attachment strength to friends is significantly lower when a new romantic relationship has begun. These results are consistent with Hypothesis 3. Further supporting this hypothesis are the findings for the Safe Haven and Secure Base functions: even though partners are present in the attachment network even in shorter relationships, only after 24 months do partners come to assume greater relevance, then reaching the same level of importance as mothers for the Secure Base function.

Similarly, the likelihood of considering partners, rather than friends, as primary figures or as full-blown attachments, increases with the length of the romantic relationship. The corresponding decrease in the likelihood of friends fulfilling these roles is so marked that for those involved in a relationship for longer than 24 months, mothers are even more likely than friends to be considered primary figures or full-blown attachments.

This reassignment pattern fits with previous research (Umemura et al., 2017; Wrzus et al., 2017), showing that partners gradually take over from friends as attachment figures, as a romantic relationship develops further, and particularly after two years. It has been demonstrated clearly that, although the impact of a romantic relationship starts to modify attachment networks even during the first few months (Fagundes and Schindler, 2012), roughly two years are needed to develop a full attachment bond with the partner (Hazan and Zeifman, 1994; Umemura et al., 2021).

#### 4.2.3 Employment

For both students and workers, we found that friends and partners are the main figures relied upon, with the exception of Secure Base (for which mothers dominate). Nevertheless, we observed several interesting employment effects regarding the relevance of partners. As mentioned earlier, workers in this sample were more likely to be in a romantic relationship than were the students. It is worth noting that although employment was associated with romantic status, the significant effects (main and interactive) of employment that we observed cannot be due to this association, as the analyses control for the effect of a given variable on the others, ruling out spurious effects.

Workers reported relying more on their partners than students (for Separation Protest and Safe Haven), and their partners were more likely to constitute a full-blown attachment figure. For students, in contrast, mothers and friends were more relevant in the network than they were for workers. These results are in line with Hypothesis 4, and add to our understanding of the changing nature of attachment networks in young adults; to our knowledge, employment status has not previously been investigated in studies of this life phase.

As already discussed in the context of romantic status, being in a romantic relationship is associated with higher reliance on the partner, with an overall increase on the composite indexes, and on the likelihood of considering the partner as primary figure or a full-blown attachment figure. Considering that workers are more frequently involved in a romantic relationship, it is not surprising that, among workers, we observed a higher number of participants naming the partner in key roles within the network.

From our results we can speculate that workers, being more likely to have a romantic partner, can be considered a “step ahead” in terms of attaining goals typical of this transitional phase of life. Consistent with this suggestion, in a cross-sectional study involving more than 2,400 young adults from Italy and Japan (Crocetti et al., 2015), it was observed that in both countries, young workers perceive young adulthood as a less uncertain phase than students do, and that they have a more structured vision of their life-goals and of their identities. At the same time, it seems that workers perceive less need to explore possible alternative identities, as compared to students.

#### 4.2.4 Interaction effects

The main effects of the demographic variables, as discussed to this point, were qualified by a number of significant interactions. These interactions highlight some of the complexities involved in the process of transfer of attachment functions.

Firstly, we found that for Proximity Seeking, men involved in a relationship for longer than 24 months rely on their friends more than women involved in a relationship for the same amount of time. This result suggests that for women, the reassignment of attachment functions from friends to partners throughout the development of romantic relationships is somewhat faster than for men. This same result was found in the longitudinal research of Umemura et al. (2018a,b), which showed that attachment preference and reliance on partner is stronger for women than for men, already after the first year of relationship involvement.

For Secure Base we found that for both male students and workers, the most relied upon figure is the mother, followed by friends and then by fathers. However, students reported relying more on their fathers than male workers, perhaps reflecting their tendency to remain financially (and possibly emotionally) dependent on them. Additionally, among those involved in a romantic relationship for less than 24 months, students rely more on their fathers than workers for the Secure Base function, although the ordering of target figure is the same for both groups (mothers, partner and friends, and then fathers). This result further suggests an association between involvement in the world of work and progress toward emancipation from the family of origin.

The final interaction effect regards attachment strength to partners, which is the highest in workers who are involved in a romantic relationship (regardless of its length), as compared to students. Among students, on the other hand, friends and partners show the highest attachment strength. This finding is in line with the previous results, and with the hypothesis that workers show an enhanced tendency to rely on partners to meet their attachment needs.

## 5 Conclusion

In conclusion, we have examined attachment networks in young adults in their twenties, and found that complex factors shape such networks. In particular, network restructuring seems related to gender, and to the development of both romantic relationships and work commitments. Hence, although attachment processes are thought to be universal, the relative strength of different figures in the attachment hierarchy during this period of transition varies according to individual, social and economic factors.

Given the current prolonged duration of the young adult phase, it is important to extend this research to later stages of life in order to understand the trajectories more fully. For example, do young adults continue their gradual loosening of ties with their families of origin (without severing them)? Or do they encounter obstacles when approaching developmental milestones, leading to renewed dependence on the family? Attachment networks can be considered one of the key expressions of affective and relational development. From this point of view, it is crucial for counselors and clinicians to keep in mind the factors that may promote or impede the progression of developmental tasks, especially within a socio-economic context that delays the achievement of certain milestones, such as leaving the family home.

### 5.1 Limitations and future directions

Some of the results of this study raise further questions, and suggest new lines of research. Among our demographic variables, employment status, in particular, deserves further investigation. Young workers are more likely than students to be involved in a romantic relationship, and at the same time, the assignment of attachment functions from friends to partners seems to progress more quickly for workers than it does among students. This result suggests that those who are working and involved in a romantic relationship are clearly carrying out the developmental goals typically associated with young adulthood, and incorporating new figures external to the family of origin in their attachment networks, particularly in terms of the romantic partner.

Thus, it could be useful to replicate this study on the attachment networks of young adults by exploring in more detail the effects of employment status. Specifically, among the broad category of ‘workers’, it could be important to examine the role of such distinctions as employment type (stable, versus temporary or occasional). Alongside this variable, future studies should consider other aspects of participants’ progress towards key developmental goals. For example, research by Ferrari et al. (2013) suggests the relevance of a trichotomous variable, “choice of leaving the family-of-origin home”: this variable compares those who do not express the intention of leaving, those who express the intention only, and those who have actually succeeded in acting on the intention. When including these variables, it would be interesting to investigate the temporal dynamics among employment status, romantic status and choice of leaving the family-of-origin home, by employing a longitudinal design with relevant statistical tests used to examine possible causal relationships.

With respect to methodology, it is important to underline that our design was cross-sectional, and cannot lead to firm conclusions about how the variables of interest may impact on attachment networks dynamically, across time. It would be informative to investigate the process of the reassignment of attachment functions by employing a longitudinal design, particularly for the temporal dynamics involved in the relationship between employment and romantic status.

Other results that suggest avenues for further investigation include gender effects. Specifically, it could be interesting to study in more detail the relationships between young male adults and their friends, which appeared to remain relevant across attachment functions, even when a romantic relationship is present (unlike for women). Specifically, we do not know if this finding is consistent across cultures. Further, we do not know whether this finding applies, regardless of where young adults reside: it could be that living with parents, with friends, or with the partner, can change the role of friends for young men.

Another interesting result concerns the relationships between young women and their mothers, which remain important even after a strong bond has been formed between the offspring and their romantic partners. In particular, it could be useful to investigate if and how this effect changes across cultures.

In our study, we did not investigate the relationships between gender identities and biological sex of participants, nor did we assess sexual orientation. Given that we found effects of gender and romantic status, it could be interesting to replicate this study within the LGBTQIA+ community, checking whether homogeneous groups of participants, differing by gender identity or sexual orientation, differ also in attachment network structure.

Finally, from a cross-cultural perspective, it is important to remember that our sample was made up entirely of Italian participants. However, similar patterns of prolonged co-residence with the family of origin have been observed in other countries as well. Indeed, a recent Eurostat dataset (Eurostat, 2022) showed that Italy ranks seventh among the European Union countries in terms of the age at which young adults leave the parental home, with an average age of 29.9 years; in Portugal, Croatia, Slovakia, Greece and Bulgaria the average age is even higher, demonstrating the relevance of this process, particularly in Central-Eastern European countries. It could be interesting to investigate young adults’ attachment networks in these countries as well.

## Data availability statement

The raw data supporting the conclusions of this article will be made available by the authors, without undue reservation.

## Ethics statement

The studies involving humans were approved by Crip University of Milan Bicocca. The studies were conducted in accordance with the local legislation and institutional requirements. The participants provided their written informed consent to participate in this study.

## Author contributions

LC: Conceptualization, Funding acquisition, Project administration, Supervision, Writing – original draft, Writing – review & editing. PA: Formal analysis, Methodology, Software, Writing – original draft, Writing – review & editing. EA: Data curation, Writing – review & editing. CC: Conceptualization, Supervision, Writing – review & editing. FC: Methodology, Supervision, Writing – review & editing. MG: Methodology, Supervision, Writing – review & editing. LM: Data curation, Writing – review & editing. DT: Methodology, Supervision, Writing – review & editing. JF: Conceptualization, Methodology, Supervision, Writing – original draft, Writing – review & editing.

## Footnotes

^1^When we indicate in the text that a group is more likely than another to consider a figure rather than another, we intend that the odd of the first group of considering that figure is larger than the odd of the second group.
